# The association of physician-clinical pharmacist collaboration frequency and experience on physician burnout: are there gender differences

**DOI:** 10.3389/fpsyt.2025.1627734

**Published:** 2025-07-28

**Authors:** Yuancheng Jiang, Hongmeng Zhang, Chuchuan Wan, Zonghao Wu, Yuankai Huang, Ennan Wang

**Affiliations:** ^1^ The Research Center of National Drug Policy and Ecosystem, China Pharmaceutical University, Nanjing, Jiangsu, China; ^2^ School of Health Policy and Management, Nanjing Medical University, Nanjing, Jiangsu, China

**Keywords:** burnout, collaboration, pharmacists, physicians, gender equity

## Abstract

**Introduction:**

Burnout is a widespread issue among physicians globally, with pronounced gender disparities observed in China. This paper aims to analyze the collaboration characteristics between clinical pharmacists and physicians in China, examine the impact of collaboration experience and frequency on physician burnout, and explore potential gender differences in these mechanisms to improve collaboration efficiency and reduce physician burnout. Collaboration experience was measured using the “Physician Experience” dimension from the Kuwait questionnaire. Collaboration frequency was assessed by asking physicians how often they collaborate with clinical pharmacists in their daily work.

**Methods:**

A cross-sectional study was conducted between July and August 2019 across 93 urban clusters in 31 Chinese provinces. The paper examined collaboration frequency and experience between physicians and clinical pharmacists in secondary and tertiary healthcare institutions, as well as physician burnout status. Data were analyzed using ordinal logistic regression models.

**Results:**

A total of 1,381 questionnaires were distributed and 1,322 were included in the analysis. The results indicate that both the collaboration frequency and experiences between physicians and clinical pharmacists are negatively correlated with burnout. Additionally, gender shows an interactive effect in the negative relationships among collaboration frequency, collaboration experiences, and burnout. Specifically, the inclusion of gender as a variable weakened the negative correlation between collaboration frequency and the cynicism sub-dimension of job burnout. At the same level of collaboration experience, female physicians reported higher levels of cynicism and reduced personal accomplishment in job burnout compared to their male counterparts.

**Conclusion:**

This paper suggests that increasing collaboration frequency between physicians and clinical pharmacists, improving the collaborative experience, and paying particular attention to female physicians’ needs can better reduce physician burnout and improve healthcare service efficiency and quality.

## 1 Introduction

Since Freudenberger introduced the concept of burnout in 1974, the phenomenon has been extensively studied. Maslach’s three-dimensional conceptualization is now widely accepted, with the Maslach Burnout Inventory-General Survey (MBI-GS) defining burnout as a syndrome characterized by emotional exhaustion, cynicism, and reduced personal accomplishment—emotional exhaustion being the core indicator (1).

Burnout is pervasive among physicians, with prevalence rates ranging from 25% to 60% (2–7). A meta-analysis by international scholars indicates that physician burnout rates may reach 80.5% (8). Empirical evidence demonstrates that physician burnout is associated with compromised patient care quality (9) and organizational disruptions, including absenteeism and turnover (10). Given these negative consequences (11, 12), physician burnout has emerged as a global health workforce concern, prompting international organizations and national governments to develop policy responses (13, 14). Research indicates that physicians burnout in China is particularly pronounced, with those practicing in secondary and tertiary hospitals experiencing more severe symptoms than their counterparts in primary care settings (15). This underscores the importance of examining burnout among physicians in the Chinese healthcare context.

Among the various theoretical models developed to understand job burnout—such as the Job Demand-Control (JD-C) model (16), the Job Demand-Control-Support (JD-C-S) model (11), the Effort-Reward Imbalance (ERI) model (17), and the Job Demand-Resource (JD-R) model (18)—this study adopts the Person–Environment Fit (P-E Fit) model as its central framework. Unlike models that primarily emphasize external job demands or imbalances, the P-E Fit model, as introduced by Maslach and Leiter (12). This model posits that burnout results not only from individual stress but also from the interaction between the individual and their environment. It is determined by the degree of fit or misfit between the individual and six areas of their work environment: workload, control, rewards, relationships, fairness, and values (1). Therefore, rather than focusing solely on stressors or resources, the P-E Fit model emphasizes the dynamic interaction between the individual and the environment. Contemporary research has further operationalized P-E Fit along four relational dimensions: person-supervisor (PS) fit, person-organization (PO) fit, person-job (PJ) fit, and person-group (PG) fit (19, 20).

A study conducted in the United States identified social support as a critical factor in job burnout among primary healthcare providers, noting that the inclusion of clinical pharmacists in healthcare teams has a positive impact on reducing burnout. Providers working alongside pharmacists to treat complex patients may reduce emotional exhaustion and affective burden, thereby decreasing burnout (21). Further research has shown that effective teamwork in healthcare settings can alleviate physician burnout, with physicians reporting lower burnout levels when they have positive collaborative experiences (22). In light of the growing demand for pharmaceutical services, the roles and responsibilities of clinical pharmacists have expanded. Their interactions with physicians have become more frequent, positioning pharmacists as integral members of the healthcare team and essential components of physicians’ work environment and interpersonal dynamics.

In parallel, gendered dynamics within healthcare organizations merit close attention. Gendered Organization Theory (23) posits that male workers are more likely to be favored within organizational structures, and gender disparities have long persisted in workplace environments. Becker’s research (24) notes that “men have an advantage over women in the labor market.” In China, the influence of Confucian ideology has reinforced traditional gender roles, with the belief that “men work outside and women manage the household” deeply embedded in society. As a result, Chinese women often face disadvantages in the workplace compared to their male counterparts (25–28). International studies (29) mention that “women are more likely to face professional crises such as distrust in male-dominated fields.” Moreover, research indicates that “female physicians often face greater challenges in balancing work and family responsibilities compared to male physicians, leading to increased work-family conflicts and stress” (30). McMurray (31) reported that female doctors are 60% more likely to experience burnout than male doctors, while Shujuan Yang’s research found that female physicians report higher levels of burnout compared to their male counterparts (32). These findings underscore the distinct impact of gender within organizational settings, particularly in the medical profession. Building on the burnout mechanisms discussed earlier, this study argues that gender differences may serve as an important moderating factor in the relationship between physician–pharmacist collaboration and burnout, and should be taken into account in the research.

However, Thomas (33), in a review of 15 studies on resident doctors, found that only four studies identified gender as a significant factor in burnout, and none suggested that female physicians are at greater risk or experience different impacts. Research by Maslach (34) found that male physicians had significantly higher depersonalization scores than their female colleagues, potentially indicating a greater predisposition to burnout. Nathalie (35) found no clear relationship between gender and burnout. These inconclusive findings suggest that the relationship between gender and burnout may not be straightforward. While existing literature indicates that gender may influence burnout, no studies have specifically examined gender as a moderating variable in this context. This study attempts to include gender as a moderating variable, aiming to further explore its moderating effect of gender on the relationship between physician collaboration and burnout.

Building on existing literature, this study proposes two hypotheses: H1: Both collaboration frequency and experience between Chinese physicians and clinical pharmacists are negatively associated with physician burnout. H2: Gender moderates the relationship between collaboration (frequency and experience) and physician burnout, such that the strength or direction of the association varies by gender. Guided by the Person-Environment (P-E) model and focusing on person-group (PG) fit this study aims to elucidate the mechanisms through which physician-pharmacist collaboration experience and frequency influence physician burnout in healthcare institutions. Additionally, it seeks to explore the moderating role of gender in this process.

Currently, research on physician-pharmacist collaboration in China is limited, and the healthcare systems in international studies differ significantly from that of China, which makes their conclusions less directly applicable. Additionally, there is a research gap regarding the relationship between collaboration experience, frequency, and physician burnout. This study aims to address this gap by offering a novel perspective on enhancing collaboration between clinical pharmacists and physicians in China. Furthermore, this research specifically examines gender differences in the relationship between collaboration experience, frequency, and physician burnout, providing valuable insights for policymakers to design targeted interventions for burnout among physicians of different genders. To achieve this, the study will integrate data on the characteristics of physician-pharmacist collaboration in China, analyze microdata from secondary and tertiary healthcare institutions, and investigate the links between collaboration experience, collaboration frequency, and physician burnout, with a focus on gender differences. This research is crucial for improving collaboration efficiency between physicians and clinical pharmacists and for reducing physician burnout.

In summary, this study offers both theoretical innovation and significant practical value, providing scientific evidence and practical recommendations for enhancing collaboration efficiency between clinical pharmacists and physicians and mitigating physician burnout in China.

## 2 Materials and methods

### 2.1 Research design and sampling

A cross-sectional survey was conducted between July and August 2019, based on informed consent and voluntary participation, to investigate the collaboration frequency and experience between physicians and clinical pharmacists, as well as physician burnout in secondary and tertiary healthcare institutions in China. In China, healthcare institutions are classified into three levels: primary healthcare institutions, secondary hospitals, and tertiary hospitals. Secondary and tertiary hospitals, which are primarily municipal, provincial, or national comprehensive hospitals, provide specialized healthcare services to a wide range of regions and play a crucial role in medical education and scientific research. Studies have indicated that burnout rates are generally lower in primary healthcare institutions, and clinical pharmacy services are predominantly offered by tertiary hospitals. Therefore, to ensure greater representativeness, the study focused on physicians working in secondary and tertiary healthcare institutions.

To enhance the representativeness of the sample and accurately reflect the working conditions of physicians in China, a multistage sampling method was employed. The sampling process involved the following steps (1): All 31 provinces (including autonomous regions and municipalities) in mainland China were included. Within each province, all prefecture-level cities or districts were categorized into three clusters—high, medium, and low—based on per capita GDP in 2018, resulting in a total of 93 city clusters (2). Within each city cluster, hospitals were selected using convenience sampling from healthcare institutions with survey authorization, ensuring that at least two tertiary hospitals were included in each cluster. A total of at least 372 hospitals were eligible for inclusion in this study (3). Within each selected hospital, physicians were recruited through convenience sampling, provided that at least two eligible physicians consented to participate and completed the survey. Eligibility criteria for physicians included possession of a medical qualification certificate, active employment in a clinical department, and prescribing authority for either outpatient or inpatient care. Physicians in non-clinical roles (e.g., laboratory technicians), those without prescribing authority (e.g. physician assistants), or those not registered at the sampled hospitals were excluded. This study aimed to survey at least 744 eligible physicians who agreed to participate.

According to epidemiological research requirements, the sample size should be 5 to 10 times the number of factors. The burnout incidence rate among physician ranges from approximately 25% to 80.5%. Considering a 10% dropout rate, the estimated sample size was at least (5×13/0.665)/0.9 = 135 cases. Ultimately, a total of 1,322 eligible physicians from 372 hospitals completed the survey and were included in the final analysis.

Convenience sampling was used at the hospital and physician levels primarily due to resource constraints and access limitations. While this approach facilitates practical implementation across diverse regions, it may introduce selection bias and limit the generalizability of results. This limitation is acknowledged as a trade-off for achieving nationwide coverage within feasible resource boundaries.

### 2.2 Questionnaire design

#### 2.2.1 Collaboration frequency

Collaboration frequency was assessed by asking physicians how often they collaborate with clinical pharmacists in their daily work. Responses were categorized as follows “Never or rarely,” “At least once a week,” and “Once a day or more.”

#### 2.2.2 Collaboration experience

Collaboration experience was measured using the “Physician Experience” dimension from the Kuwait questionnaire (36). Which was originally designed to evaluate physicians’ perceptions and experiences of collaboration with pharmacists in the Kuwaiti healthcare system. The original scale was validated within hospital settings in Kuwait and demonstrated good psychometric properties, particularly in measuring interprofessional collaboration between physicians and clinical pharmacists. In this study, the instrument was translated and adapted for use in the Chinese healthcare context, with appropriate cultural and linguistic modifications to ensure relevance. Respondents rated their agreement with each item using a 5-point Likert scale (1 = “Strongly disagree” to 5 = “Strongly agree”) based on their practical collaboration experience with clinical pharmacists. The adapted version yielded a Cronbach’s α coefficient of 0.874 and a KMO value of 0.881, indicating strong internal consistency and suitability for factor analysis in the Chinese sample.

#### 2.2.3 Burnout

The Chinese version of the 15-item Maslach Burnout Inventory General Survey (MBI-GS) was used, with permission obtained through official channels. In 2002, Li Chaoping (37) conducted an exploratory factor analysis on the 16 items of the MBI-GS using principal component extraction and orthogonal rotation, finding a high cross-load for one item. After deleting this item, a second factor analysis confirmed that the adjusted structure of the MBI-GS was identical to the original, suggesting that the instrument maintains good construct validity in China. The internal consistency coefficients for emotional exhaustion, cynicism, and reduced personal accomplishment were 0.88, 0.83, and 0.8244, respectively. The questionnaire used a 7-point Likert scale, where 0 represented “Never” and 6 represented “Very frequently.” It included 5 questions on emotional exhaustion, 4 questions on cynicism, and 6 questions on personal accomplishment. Emotional exhaustion and cynicism were scored positively, while personal accomplishment was scored negatively. The total burnout score was calculated as: Burnout Total Score = 0.4 × Average Emotional Exhaustion Score + 0.3 × Average Cynicism Score + 0.3 × Average Personal Accomplishment Score. Although not part of the original MBI-GS manual, the approach has been used in Chinese healthcare burnout research to yield more nuanced scores. The total score ranges from 0 to 1.49 (No burnout), 1.50 to 3.49 (Moderate burnout), and 3.50 to 6 (Severe burnout). The KMO value of the questionnaire was 0.910, indicating it is highly suitable for factor analysis.

Specifically, we conducted an exploratory factor analysis (EFA) using principal component extraction with varimax rotation (Kaiser normalization). The analysis yielded four factors, with acceptable factor loadings for each item. Factor 1 included 6 items related to achievement frustration (loadings ranged from 0.777 to 0.895); Factor 2 included 5 items related to emotional exhaustion (loadings from 0.744 to 0.887); Factor 3 contained 4 items representing burnout; and Factor 4 included items related to medication consultation and collaboration, with loadings above 0.522. The factor structure was consistent with theoretical expectations.

#### 2.2.4 Control variables

To control for potential confounding factors, the study incorporated both personal variables (e.g., gender, marital status, number of children, education level, and region) and organizational factors (e.g., years of service, hospital level, hospital type, department, technical title, administrative position, and frequency of involuntary attendance). These variables were considered as they may influence the extent of physician burnout.

The variables were measured as follows: hospital type、marital status、professional title、education level、department、administrative position、region and frequency of working with illness in the past year were treated as categorical variables, with predefined codes assigned to each category; age was treated as a continuous variable. These operational definitions were adopted to enhance measurement consistency and ensure analytical reproducibility when evaluating their potential influence on physician burnout.

### 2.3 Data collection

In this study, university students with professional medical backgrounds and research training were selected as interviewers. These interviewers conducted random interviews with physicians from the sample hospitals to collect relevant data. At least five doctors from each sample hospital agreed to participate in the survey. Prior to the formal survey, the interviewers identified the hospitals’ non-working hours and, with verbal permission from hospital directors or deputy directors, entered the facilities. The interviewers provided an oral explanation of the survey’s background, content, and purpose to the physicians and administered the questionnaire to those who agreed to participate and signed an informed consent form.

During the survey, the interviewers used mobile electronic devices equipped with survey software to administer the questionnaires. They explained how to answer the questions, read the items and answer choices aloud, and recorded the respondents’ verbal responses. Results from a preliminary survey indicated that this approach was feasible and that the survey demonstrated high reliability and validity. Given the relatively short length of the questionnaire, respondents did not experience survey fatigue, ensuring the quality of the data collected. All research was conducted in an undisturbed environment, and interviewers refrained from expressing any opinions or biases regarding the study content before or during the survey.

Although oral administration by trained interviewers helped ensure comprehension and data completeness, it may have introduced social desirability or interviewer bias. To minimize such effects, interviewers adhered to a standardized script, maintained neutral tone, and conducted interviews in private settings without institutional observers.

### 2.4 Data analysis and model testing

The electronic survey data were imported into SPSS 26.0 for initial processing, and data verification was independently conducted by two researchers. In this study, physician burnout served as the dependent variable, while collaboration frequency and collaboration experience were the primary independent variables. Demographic and work-related characteristics were included as control variables. Descriptive statistics were calculated for all relevant variables, as presented in **Table 1**. This study examined the relationship between physician-pharmacist collaboration frequency and experience and physician burnout, and to explore whether there were gender differences, the study investigated the interaction effects of gender on burnout and its three sub-dimensions. Ordinal logistic regression analysis was conducted using STATA 17.0, with statistical significance defined as p < 0.05. To address potential endogeneity and evaluate the robustness of the findings, the study included multicollinearity and robustness checks using STATA 17.0. A robustness test was performed by incorporating “administrative position” as an additional independent variable to determine whether the model’s results remained stable. Additionally, the study tested whether there were any multicollinearity issues among the independent variables.

**Table 1:** Demographic characteristics of physicians and burnout level (n=1322).

Characteristic	Frequency/Proportion (n/%)
Hospital level	
Secondary	697 (52.72%)
Tertiary	625 (47.28%)
Hospital type	
General Hospital	1008 (76.25%)
Specialty Hospital	63 (4.77%)
Traditional Chinese Medicine	196 (14.83%)
Other	55 (4.16%)
Gender	
Male	698 (52.8%)
Female	624 (47.2%)
Marital status	
Unmarried	222 (16.79%)
Married	1093 (82.68%)
Other (Divorced, Widowed, etc.)	7 (0.53%)
Age	
37.81 (Mean)	9.24 (Standard Deviation)
Professional title	
Junior	457 (34.57%)
Intermediate	532 (40.24%)
Associate Senior	258 (19.52%)
Senior	75 (5.67%)
Education level	
Below Bachelor’s	115 (8.7%)
Bachelor’s	865 (65.43%)
Master’s	309 (23.37%)
Doctorate	33 (2.5%)
Unpaid overtime frequency in the past year	
Never	558 (42.2%)
Once	355 (26.9%)
2–5 times	318 (24.1%)
More than 5 times	91 (6.9%)
Department	
Internal Medicine	438 (33.13%)
Surgery	287 (21.71%)
Obstetrics and Gynecology	81 (6.13%)
Pediatrics	85 (6.43%)
Otolaryngology	108 (8.17%)
Dermatology	37 (2.8%)
Traditional Chinese Medicine	47 (3.56%)
Other	239 (18.08%)
Administrative position	
Department Head	141 (10.7%)
Deputy Department Head	164 (12.4%)
General Physician	1017 (76.9%)
Region	
Eastern	488 (36.9%)
Central	359 (27.2%)
Western	475 (35.9%)
Burnout level	
No	514 (38.88%)
Moderate	776 (58.7%)
Severe	32 (2.42%)
Emotional exhaustion	
10.04 (Mean)	5.51 (Standard Deviation)
Cynicism	
4.46 (Mean)	3.96 (Standard Deviation)
Reduced personal accomplishment	
11.72 (Mean)	7.59 (Standard Deviation)

### 2.5 Ethics

This study was approved by the Ethics Committee of China Pharmaceutical University (No.CPU2019015). The study was conducted after obtaining informed consent from the participants. Participants had the right to refuse to answer any questions or withdraw from the study at any time at any stage of the study. We certify that the study was performed in accordance with the 1964 declaration of HELSINKI and later amendments. To ensure confidentiality, no personally identifiable information was collected during interviews. Data were anonymized upon collection, and access was restricted to the research team. All digital data were stored in password-protected devices following institutional data protection protocols.

## 3 Results

### 3.1 Demographic characteristics of physicians and job burnout

A total of 1,500 questionnaires were distributed, and 1,381 questionnaires were returned, yielding a response rate of 92.1%. After excluding invalid questionnaires, the final valid sample size was 1,322.

As shown in **Table 1**, 47.28% of physicians were from tertiary hospitals, and 52.72% from secondary hospitals. Regarding professional titles, 34.57% held junior titles, 40.24% intermediate, 19.52% associate senior, and 5.67% senior titles. The majority (76.9%) had no administrative role. Physicians were primarily from internal medicine departments, and participants were distributed across the eastern, central, and western regions of China.

Over the past 12 months, more than half of the physicians reported situations where they were unable to take leave and had to work while ill. Regarding job burnout, more than half (61.12%) of the physicians exhibited signs of burnout, with 58.70% experiencing moderate burnout and 2.42% indicating severe burnout. Specifically, emotional exhaustion scored 10.04 ± 5.51, cynicism scored 4.46 ± 3.96, and reduced personal accomplishment scored 11.72 ± 7.59 (see **Table 1**). Burnout classification was based on the composite score derived from all three sub-dimensions (emotional exhaustion, cynicism, and reduced personal accomplishment), using the weighted formula described in the Methods section. Therefore, reported prevalence rates reflect aggregated burnout levels, not isolated dimension thresholds.

### 3.2 Regression analysis

#### 3.2.1 Main effects

Ordinal logistic regression analysis was conducted to examine the relationship between physician-pharmacist collaboration experience and collaboration frequency (independent variables) and job burnout (dependent variable), while controlling for factors such as age, gender, marital status, and other relevant variables. The results indicated that both collaboration frequency (95% CI: -0.81 to -0.31, p < 0.001)and collaboration experience (95% CI: -0.60 to -0.12, p < 0.001) were negatively associated with job burnout (see **Table 2**). Additionally, control variables such as age, frequency of involuntary attendance, department, and administrative position also had significant effects on the level of physician burnout. The study employed ordinal logistic regression and tested the proportional odds assumption, collinearity, and robustness. The results indicated that the proportional odds assumption was satisfied (χ² = 101.208, df = 120, p = 0.892). The results of the collinearity test were satisfactory, with the mean Variance Inflation Factor (VIF) values being 1.56 and 1.55 (<3.25, <3.29), indicating that there were no multicollinearity issues among the independent variables. The average Variance Inflation Factor (VIF) values were 1.56 and 1.55, well below the commonly accepted threshold of 5, indicating no multicollinearity among predictors (38).” A robustness test was conducted by adding a new variable, “administrative position”, to the model. The specific administrative positions included were department head, deputy department head, and general physician. After incorporating this new variable, all key coefficients (including those of collaboration frequency and experience) retained their direction and remained statistically significant, suggesting the findings were stable.

**Table 2:** Ordinal logistic regression results for factors related to physician burnout.

Job burnout	COEF.	[95%Conf.	Interval]
Collaboration Experience	-0.56***	-0.81	-0.31
Hospital Type: General Hospital (Control Group)			
Tertiary	0.15	-0.09	0.39
Specialized Hospital	-0.31	-0.86	0.23
Traditional Chinese Medicine Hospital	-0.22	-0.54	0.10
Other	-0.23	-0.79	0.34
Marital Status: Unmarried (Control Group)			
Married	-0.15	-0.53	0.23
Other	-1.27	-2.99	0.45
**Age**	-0.03***	-0.05	-0.01
Professional Title: Junior Title (Control Group)			
Intermediate Title	0.08	-0.24	0.39
Associate Senior Title	0.15	-0.29	0.60
Senior Title	-0.21	-0.85	0.43
Education Level: Below Bachelor's Degree (Control Group)			
Bachelor's Degree	0.17	-0.25	0.58
Master's Degree	0.20	-0.28	0.67
Doctorate	-0.11	-0.95	0.73
Frequency of Working with Illness in the Past Year: Never (Control Group)			
Once	0.27*	-0.01	0.55
2-5 Times	0.42***	0.13	0.72
More than 5 Times	1.17***	0.66	1.69
Department: Internal Medicine (Control Group)			
Surgery	0.16	-0.1523791	0.48
Obstetrics and Gynecology	-0.11	-0.61	0.39
Pediatrics	-0.12	-0.61	0.37
Otorhinolaryngology	-0.08	-0.52	0.36
Dermatology	-0.95***	-1.65	-0.25
Traditional Chinese Medicine	-0.65**	-1.28	-0.02
Other	-0.09	-0.43	0.24
Administrative Position: Department Head (Control Group)			
Deputy Department Head	-0.53**	-1.02	-0.05
General Doctor	-0.16	-0.58	0.26
Region: Eastern (Control Group)			
Central	-0.16	-0.45	0.12
Western	0.06	-0.21	0.33
/cut1	-2.34	-3.35	-1.33
/cut2	2.00	0.96	3.05
	Number of obs		1,322
	LR chi2(28)		101.77
	Prob > chi2		0
	Pseudo R2		0.05

***p< 0.01 **p<0.05 *p<0.1.

**Table:** 

Job burnout	COEF.	[95%Conf.	Interval]
Collaboration Frequency: Never/Rarely (Control Group)			
At least once a week	-0.36***	-0.60	-0.12
Once a day or more	-0.51**	-0.90	-0.12
Hospital Level: Secondary (Control Group)			
Tertiary	0.14	-0.10	0.38
Hospital Type: General Hospital (Control Group)			
Specialized Hospital	-0.25	-0.80	0.30
Traditional Chinese Medicine Hospital	-0.21	-0.52	0.11
Other	-0.22	-0.78	0.34
Marital Status: Unmarried (Control Group)			
Married	-0.13	-0.51	0.25
Other	-1.21	-2.92	0.51
**Age**	-0.03***	-0.05	-0.01
Professional Title: Junior Title (Control Group)			
Intermediate Title	0.12	-0.20	3
Associate Senior Title	0.17	-0.27	0.62
Senior Title	-0.17	-0.81	0.47
Education Level: Below Bachelor's Degree (Control Group)			
Bachelor's Degree	0.18	-0.23	0.60
Master's Degree	0.20	-0.28	0.68
Doctorate	-0.08	-0.92	0.77
Frequency of Working with Illness in the Past Year: Never (Control Group)			
Once	0.30**	0.02	0.57
2-5 Times	0.45***	0.16	0.74
More than 5 Times	1.17***	0.65	1.68
Department: Internal Medicine (Control Group)			
Surgery	0.12	-0.19	0.44
Obstetrics and Gynecology	-0.19	-0.69	0.31
Pediatrics	-0.14	-0.62	0.35
Otorhinolaryngology	-0.07	-0.51	0.36
Dermatology	-0.94**	-1.65	-0.24
Traditional Chinese Medicine	-0.49	-1.12	0.13
Other	-0.10	-0.43	0.23
Administrative Position: Department Head (Control Group)			
Deputy Department Head	-0.48*	-0.96	0.004
General Doctor	-0.13	-0.55	0.29
Region: Eastern (Control Group)			
Central	-0.16	-0.45	0.12
Western	0.08	-0.19	0.35
/cut1	-1.60	-2.54	-0.67
/cut2	2.72	1.74	3.70
	Number of obs		1,322
	LR chi2(29)		93.22
	Prob > chi2		0
	Pseudo R2		0.05

***p< 0.01 **p<0.05 *p<0.1.

#### 3.2.2 Interaction effects

Gender was included in the model as an interaction term to explore its potential moderating role. The interaction effects were further examined by incorporating interaction terms between gender and the key independent variables. Specifically, to assess the potential moderation effect of gender, interaction terms—collaboration experience × gender and collaboration frequency × gender—were added to the regression models. Job burnout was conceptualized as comprising three sub-dimensions: emotional exhaustion, cynicism, and reduced personal accomplishment. These dimensions were analyzed separately (see **Table 3**). Collaboration experience was found to be significantly associated with lower levels across all three sub-dimensions. In contrast, collaboration frequency was significantly negatively associated only with cynicism, while its associations with emotional exhaustion and reduced personal accomplishment did not reach statistical significance. The results suggest that the associations between collaboration experience and collaboration frequency with job burnout may differs by gender. Specifically, female physicians reported higher levels of burnout, cynicism, and reduced personal accomplishment compared to their male counterparts, even at the same level of collaboration experience. Furthermore, the inclusion of gender as a variable weakened the negative correlation between collaboration frequency and the cynicism sub-dimension of job burnout. These findings imply that although collaboration with clinical pharmacists is generally associated with lower levels of burnout, this association may be less pronounced among female physicians, particularly with regard to cynicism (see **Table 3**). Such gender differences may be partly attributable to organizational or cultural factors. For example, female physicians may encounter greater work-life conflict and experience lower levels of institutional support, which could potentially weaken the protective association between interprofessional collaboration and burnout-related outcomes.

**Table 3:** Results for interaction effects.

	Model 1	Model 2	Model 3	Model 4
	Job burnout	Emotional exhaustion	Cynicism	Reduced personal accomplishment
Collaboration Experience	-0.21***	-0.61*	-0.76***	-2.18***
Gender	-0.05	-0.55*	-0.45**	0.46
Interaction term	0.18*	0.17	0.90**	1.89**
_cons	2.36***	11.20***	6.47***	19.50***

	Model 1	Model 2	Model 3	Model 4
	Job burnout	Emotional exhaustion	Cynicism	Reduced personal accomplishment
Collaboration Frequency	-0.0**	-0.21	-0.45***	-0.31
Gender	-0.04	-0.52*	-0.40*	0.59
Interaction term	0.08	0.67	0.95***	-0.81
_cons	2.04***	10.30***	5.39***	16.23***

***p< 0.01, **p<0.05, *p<0.1.

However, the moderation effects were statistically significant primarily for the cynicism and emotional exhaustion dimensions, and weaker or non-significant for reduced personal accomplishment. These nuanced differences should be interpreted with caution.

## 4 Discussion

### 4.1 Demographic characteristics of physicians and job burnout levels

The study found that both collaboration frequency and collaboration experience between physicians and clinical pharmacists were significantly negatively correlated with physician burnout. Physicians who collaborated “at least once a week” or “once a day or more” reported significantly lower burnout levels than those who collaborated “never or rarely”. From the perspective of the Person-Environment Fit (PG Fit) theory (1), higher collaboration frequency may indicate better communication, clearer exchange of expectations, and fewer misunderstandings or conflicts between physicians and pharmacists (39). Higher collaboration frequency reflects better communication, clearer exchange of expectations, and fewer misunderstandings or conflicts between physicians and pharmacists (40). Additionally, shared goals and values are central to PG fit. In healthcare settings, shared goals and values may contribute to more frequent collaboration between physicians and pharmacists (41). Physicians and pharmacists working together toward high-quality patient care may foster alignment, which in turn is associated with lower burnout levels (42). Similarly, more positive collaboration experiences were associated with lower perceived burnout. Collaboration experience reflects the quality of professional interactions. Positive experiences often involve mutual respect and support (43). Mutual respect between physicians and pharmacists can serve as a psychological resource that buffers against stress (44, 45). In a positive collaboration experience, physicians and pharmacists are able to recognize and support each other’s professional roles and contributions, engage in joint decision-making (46), and establish trust and effective communication (47). These factors are linked to increased job satisfaction and reduced burnout from role disrecognition (48). Furthermore, when faced with challenges in medical work, higher collaboration frequency and positive collaboration experiences between physicians and pharmacists can form a synergistic association. The expertise of clinical pharmacists in drug management (49) helps distribute workload (50), while both parties work together to find solutions, avoid medical risks (45), and help manage work-related stress and burnout (51).

It is important to note that, due to the cross-sectional nature of this study, the observed associations between collaboration and burnout do not imply causality. Future longitudinal or experimental research designs are needed to establish the direction and causality of these effects. Our research results suggest that collaboration between physicians and clinical pharmacists in China fosters a positive work environment and enhances professional interactions, effectively reducing burnout among Chinese physicians. Therefore, policymakers and healthcare administrators can attempt to further improve the collaborative environment between physicians and clinical pharmacists in subsequent efforts, enhance the quality of their collaboration, and thus reduce the risk of physician burnout (52). Policymakers and hospital administrators can implement targeted interventions to enhance collaboration environments. These may include structured interprofessional training programs, team-based performance evaluations, and regular joint case reviews between physicians and pharmacists.

In addition, the study found that male physicians experienced lower overall burnout, particularly in the cynicism sub-dimension, compared to female physicians, largely due to more favorable collaboration experiences. Meanwhile, under the same collaboration frequency, female physicians perceived higher levels of cynicism than their male counterparts. A recent study (32) found that female doctors are more likely than male doctors to take on more work, and leadership roles are primarily held by male doctors (53). Therefore, female physicians showed higher susceptibility to cynicism and reduced personal accomplishment compared to their male colleagues, which aligns with our findings. Despite increasing independence among women, female physicians in certain healthcare environments often encounter distrust, making it more difficult for them to experience positive collaboration (54, 55). These factors may contribute to the development of burnout syndrome. These gender disparities may stem from broader sociocultural and institutional dynamics in China. For example, traditional gender norms may expect women to shoulder more family responsibilities, compounding professional stress (56). Organizational cultures in Chinese hospitals often favor male physicians in leadership and decision-making roles, further limiting female physicians’ access to influential collaborative networks (57). This social marginalization may exacerbate feelings of cynicism and reduce personal accomplishment. Given the moderating effect of gender on the negative relationship between collaboration frequency, experience, and job burnout, hospital administrators should adopt flexible strategies to provide additional care and support for female medical staff, thereby reducing the risk of burnout among female physicians. To support female physicians, institutions could establish mentorship programs, offer flexible scheduling, and ensure equal participation in leadership and decision-making forums.

Additionally, the study observed that age is negatively associated with job burnout. Several studies have concluded that burnout decreases with age (58, 59), with younger or less experienced individuals being more susceptible to burnout (15, 37, 60, 61). These studies are consistent with our findings. Younger physicians tend to experience higher levels of burnout, possibly due to a lack of experience and difficulty in developing effective coping strategies (62). As they gain experience over time, most physicians accumulate sufficient experience to manage burnout effectively.

This study shows that involuntary attendance—regardless of frequency—significantly positively affects physician burnout. Working while unwell is often referred to as presenteeism (63), which has been shown in previous studies to be associated with increased burnout (64). Van Waeyenberg (65) distinguishes between voluntary and involuntary attendance, with the latter being driven by external pressures or obligations. Involuntary attendance is positively correlated with burnout, a finding that aligns with our results. In China, clinical physicians face a severe shortage of doctors and a high number of patients, making it understandable that many physicians work while ill due to professional ethics. At the same time, physicians in China, influenced by performance evaluations, may feel obligated to work despite poor health, further contributing to burnout.

The study also found that dermatology and traditional Chinese medicine physicians in China reported lower burnout levels than physicians from other departments. Currently, there is limited research on department-specific burnout both domestically and internationally. In China, many patients are less likely to seek care in dermatology or TCM, potentially resulting in lower patient volumes and reduced work pressure, which may, in turn, decrease the risk of burnout. Apart from lower patient volume, other possible explanations include different patient expectations, lower clinical urgency, or departmental cultures that emphasize holistic care and patient rapport. These aspects may foster a more manageable work environment and reduce burnout.

Furthermore, the study that clinicians with intermediate titles were less likely to experience burnout compared to those with senior titles. In China’s healthcare system, senior physicians often shoulder greater responsibilities and pressures, such as overseeing daily operations and ensuring the quality and safety of medical services. These additional duties can increase their workload, disrupt work-life balance, and contribute to higher levels of burnout. Similarly, physicians with intermediate titles may be positioned to benefit from professional stability without the heavy administrative and supervisory burdens shouldered by senior physicians. They often have more predictable schedules and less institutional accountability, which may buffer against burnout.

### 4.2 Strengths and limitations

This study has three key strengths. First, it is the first to explore the interaction effect of gender on the relationship between physician-pharmacist collaboration frequency and experience and physician burnout, addressing a gap in the existing literature. Second, the large sample size enhances the statistical power of the findings, enabling a more nuanced analysis of the relationship between physician-pharmacist collaboration and burnout. Finally, the study specifically examines China’s social and cultural context, providing valuable insights into the mechanisms of collaboration and burnout among Chinese clinical physicians and pharmacists. This context-specific approach increases the applicability of the findings to China and offers valuable guidance for policy development.

Despite the significant results, there are some limitations. First, as a cross-sectional study, it is difficult to track the changes and development of the relationship between collaboration frequency, experience, and physician burnout over time, and causal inferences cannot be drawn (66). Future studies employing longitudinal designs could provide a clearer understanding of the dynamic relationships between these variables. Second, while the questionnaire used in this study represents an attempt to adapt an international tool to the Chinese context, cultural differences may still pose challenges. Thus, further research is needed to assess the generalizability of these findings across different regions or cultural settings. Third, data collection relied on self-reported recall, which may introduce bias. Lastly, although the study controlled for variables such as marital status, age, department, and involuntary attendance, the relationship between collaboration frequency, experience, and burnout is complex, and other unexamined variables may also influence these outcomes. Future research should include additional relevant variables to provide a more comprehensive and rigorous exploration of these relationships.

Future studies could benefit from longitudinal or mixed-method designs to trace the dynamic interplay of collaboration and burnout over time. Incorporating qualitative interviews would help uncover nuanced mechanisms behind physician-pharmacist interactions. Additionally, future research should explore organizational culture, leadership styles, and specific workload measures as potential mediating or moderating variables.

## 5 Conclusion

This study demonstrates that increasing the physician-pharmacist collaboration frequency, enhancing collaboration experience, and paying special attention to female physicians can more effectively reduce physician burnout, improve healthcare efficiency, and enhance service quality. The findings offer valuable insights into physician-pharmacist collaboration and physician burnout, providing a useful reference for policymakers and hospital administrators in China to address physician burnout and promote the development of effective physician-pharmacist team collaboration.

## Data Availability

The datasets presented in this article are not readily available because The datasets used and/or analyzed during the current study are available from the corresponding author on reasonable request. Requests to access the datasets should be directed to hyk@cpu.edu.cn.
